# Molecular Determinants of Resistance Activation and Suppression by *Phytophthora infestans* Effector IPI-O

**DOI:** 10.1371/journal.ppat.1002595

**Published:** 2012-03-15

**Authors:** Yu Chen, Zhenyu Liu, Dennis A. Halterman

**Affiliations:** 1 Department of Plant Pathology, University of Wisconsin-Madison, Madison, Wisconsin, United States of America; 2 U.S. Department of Agriculture-Agricultural Research Service, Vegetable Crops Research Unit, Madison, Wisconsin, United States of America; Virginia Polytechnic Institute and State University, United States of America

## Abstract

Despite intensive breeding efforts, potato late blight, caused by the oomycete pathogen *Phytophthora infestans*, remains a threat to potato production worldwide because newly evolved pathogen strains have consistently overcome major resistance genes. The potato *RB* gene, derived from the wild species *Solanum bulbocastanum*, confers resistance to most *P. infestans* strains through recognition of members of the pathogen effector family IPI-O. While the majority of IPI-O proteins are recognized by RB to elicit resistance (e.g. IPI-O1, IPI-O2), some family members are able to elude detection (e.g. IPI-O4). In addition, IPI-O4 blocks recognition of IPI-O1, leading to inactivation of RB-mediated programmed cell death. Here, we report results that elucidate molecular mechanisms governing resistance elicitation or suppression of RB by IPI-O. Our data indicate self-association of the RB coiled coil (CC) domain as well as a physical interaction between this domain and the effectors IPI-O4 and IPI-O1. We identified four amino acids within IPI-O that are critical for interaction with the RB CC domain and one of these amino acids, at position 129, determines hypersensitive response (HR) elicitation *in planta*. IPI-O1 mutant L129P fails to induce HR in presence of RB while IPI-O4 P129L gains the ability to induce an HR. Like IPI-O4, IPI-O1 L129P is also able to suppress the HR mediated by RB, indicating a critical step in the evolution of this gene family. Our results point to a model in which IPI-O effectors can affect RB function through interaction with the RB CC domain.

## Author Summary

The potato late blight pathogen, *Phytophthora infestans*, is able to rapidly evolve to overcome resistance genes. The pathogen accomplishes this by secreting an arsenal of proteins, termed effectors, that function to modify host cells. Although hundreds of candidate effectors have been identified in *P. infestans*, their roles in pathogenicity or virulence remains basically unknown. Our results showed that one of these effectors functions to turn off resistance mediated by the potato gene RB. This effector accomplishes this by directly interacting with RB, which likely modifies its ability to turn on host resistance. Further molecular analysis identified two amino acids within the effector that determine interaction, which can assist in developing appropriate disease control strategies.

## Introduction

Plant resistance to microbial pathogens is a complex process and includes a variety of constitutive and inducible defense mechanisms [1]. Recognition and response to microbes by plants involve multiple layers of defense. The first, basal defense, relies on the recognition of conserved microbial associated molecular patterns by host receptors [2]. However, the basal defense response can be suppressed by pathogen proteins, termed effectors, that are delivered into the apoplast or plant cell cytoplasm, resulting in effector triggered susceptibility [3]–[5]. Plants have therefore evolved a second layer of defense, called effector triggered immunity (ETI), in which host protein receptors recognize the presence of pathogen effectors and elicit responses to inhibit colonization [6]. ETI relies on resistance (R) proteins to directly or indirectly recognize the presence of specific effector molecules and activate resistance signaling.

The majority of plant R proteins have nucleotide binding (NB) and leucine-rich repeat (LRR) motifs, and can be divided into two sub-classes based on a variable N-terminal domain, which typically contains coiled-coil (CC) motifs or includes homology to the Toll-interleukin-1 receptor (TIR) [7]. Regardless of the motif present, the N-terminal domains of plant NB-LRR proteins are proposed to initiate resistance signaling or pathogen recognition [8]–[14]. The interaction and coordinated activity of these different domains is likely required for activation of plant NB-LRR proteins and the signaling needed to elicit resistance responses [15]–[17].

The mechanisms of pathogen effector perception by R proteins include both direct and indirect protein interactions. The R proteins Pi-ta [18], RRS1-R [19], N [17], L5/L6 [20], M [21], and RPP1 [22] all physically interact with their corresponding effectors. Alternatively, plant R proteins can indirectly recognize the presence of pathogen effectors by monitoring target host cellular proteins [23]. Through monitoring the integrity of host targets, R proteins detect effectors indirectly, which explains how relatively conserved plant R proteins can detect highly varied pathogen effectors [23].

Potato and tomato late blight is caused by the oomycete pathogen *Phytophothora infestans* (Mont.) de Bary. The late blight *R* gene, *RB* (also known as *Rpi-blb1*), from the wild potato species *S. bulbocastanum*, encodes a CC-NB-LRR protein and confers broad-spectrum, partial resistance to most strains of the pathogen due to the almost ubiquitous presence of the corresponding effector IPI-O [24]–[29]. IPI-O is a multigene effector family and the IPI-O locus can be extremely variable between pathogen strains [24], [25], [30]. IPI-O belongs to the class of *Phytophthora* effectors with a highly conserved N-terminal RXLR motif and a C-terminal W motif [24], [31]–[34]. IPI-O variants have been divided into three classes based on diversity of their deduced amino acid sequences [24], [25]. Class I variants (e.g. IPI-O1), which are found in the majority of *P. infestans* isolates, are recognized by *RB*, while class III variants (e.g. IPI-O4) are not [26]. In addition, *P. infestans* strains lacking a class I IPI-O are virulent on plants carrying *RB* [24], and *P. infestans* strains with class III variants are more aggressive on plants with *RB* [25]. Interestingly, IPI-O4 not only eludes detection by *RB*, but is also capable of inhibiting the HR elicited IPI-O1 [25].

In the present study, we investigated intra- and intermolecular interactions of the RB protein using specific R protein domains and IPI-O variants. We identified amino acids that play a key role not only in the interaction between the RB CC domain and IPI-O4, but also in elicitation or suppression of an *RB*-mediated HR. Our findings suggest a model in which IPI-O4 is able to affect RB function through interaction with the CC domain, possibly disrupting interactions that would otherwise lead to R protein activation.

## Results

### RB CC domain self-associates and physically interacts with IPI-O1 and IPI-O4

**Figure 1:**
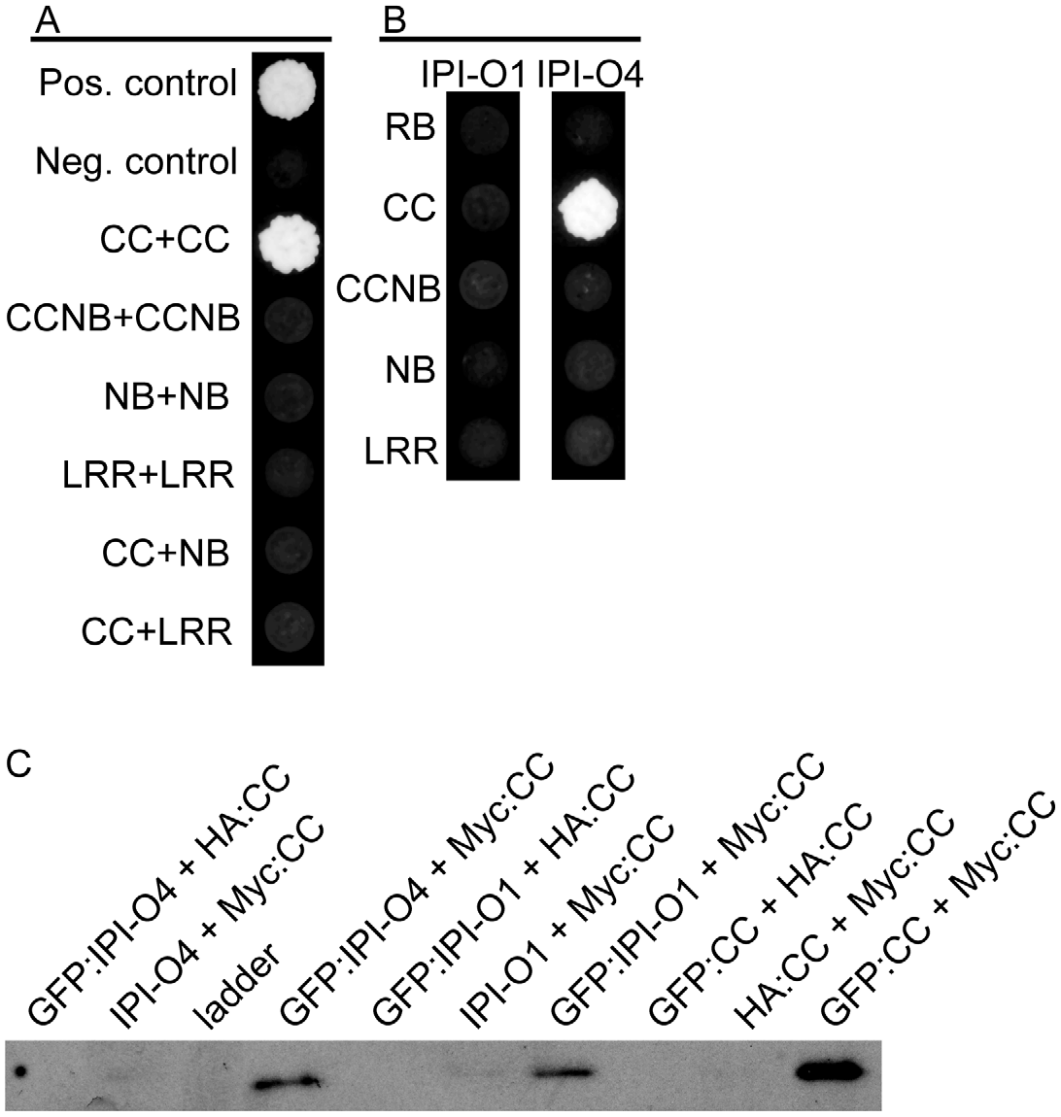
Inter- and intramolecular interactions between RB protein domains and IPI-O effectors. A) Yeast two-hybrid interactions between domains of RB or domain self-association; B) Interactions between domains of RB and IPI-O1 or IPI-O4; C) Results of co-immunoprecipitation of RB CC domain with IPI-O fusion proteins. The indicated protein combinations were expressed in *N. benthamiana* leaves and total proteins were incubated with green fluorescent protein (GFP) antibody and agarose beads. Precipitated proteins were detected using Myc-tag antibody. Proteins with no fusion or with a hemagglutinin (HA) tag were used as negative controls.

To investigate physical interactions between RB domains, pair-wise combinations of protein domains were assayed using a directed yeast two-hybrid interaction assay. Our results showed that none of the separate or fused domains of RB interacted with dissimilar domains (Figure 1A, Figure S1). Self-association of the CC domain was observed, but no self-association was detected among the NB, LRR or CCNB domains (Figure 1A). To study whether recognition of IPI-O1 by RB involves a direct R protein-effector interaction, associations between IPI-O1 or IPI-O4 with the RB CC, NB, LRR, and CCNB domains and full length RB were tested. A physical interaction was observed between the CC domain of RB and IPI-O4 (Figure 1B). However, no such interaction was found between the CC domain and IPI-O1 in yeast. No interactions between IPI-O1 or IPI-O4 with any other RB domain was observed. Also, no interaction between IPI-O1 and IPI-O4 was observed, however an interaction between IPI-O4 molecules was detected, indicating possible oligomerization of this effector (Figure S1). Protein blotting showed that all of the IPI-O and RB proteins in the pSOS expression vector were stable in yeast (Figure S2).

We verified the yeast two-hybrid data using co-immunoprecipitation in *Nicotiana benthamiana* leaves. Expression of versions of the RB CC domain, IPI-O1, and IPI-O4 fused with green fluorescent protein (GFP) showed proteins of the expected sizes in leaves after detection with GFP antibodies, although some degradation products were observed (Figure S3). Compared to GFP:RBCC and GFP:IPI-O4, full-length GFP:IPI-O1 protein only accumulated to low levels indicating that full-length versions of this protein were present, but possibly unstable. No degradation products of the RB CC domain were observed when proteins were expressed as fusions with a Myc-tag (Figure S3B). Co-immunoprecipitation confirmed oligomerization of the RB CC domain and the interaction between the RB CC domain and IPI-O4 (Figure 1C). In contrast to the yeast two-hybrid results, we also detected IPI-O1 after precipitation with the RB CC domain, indicating *in planta* interaction between these proteins.

### IPI-O1 and IPI-O4 interact with RB CC fragments from wild potato

**Figure 2:**
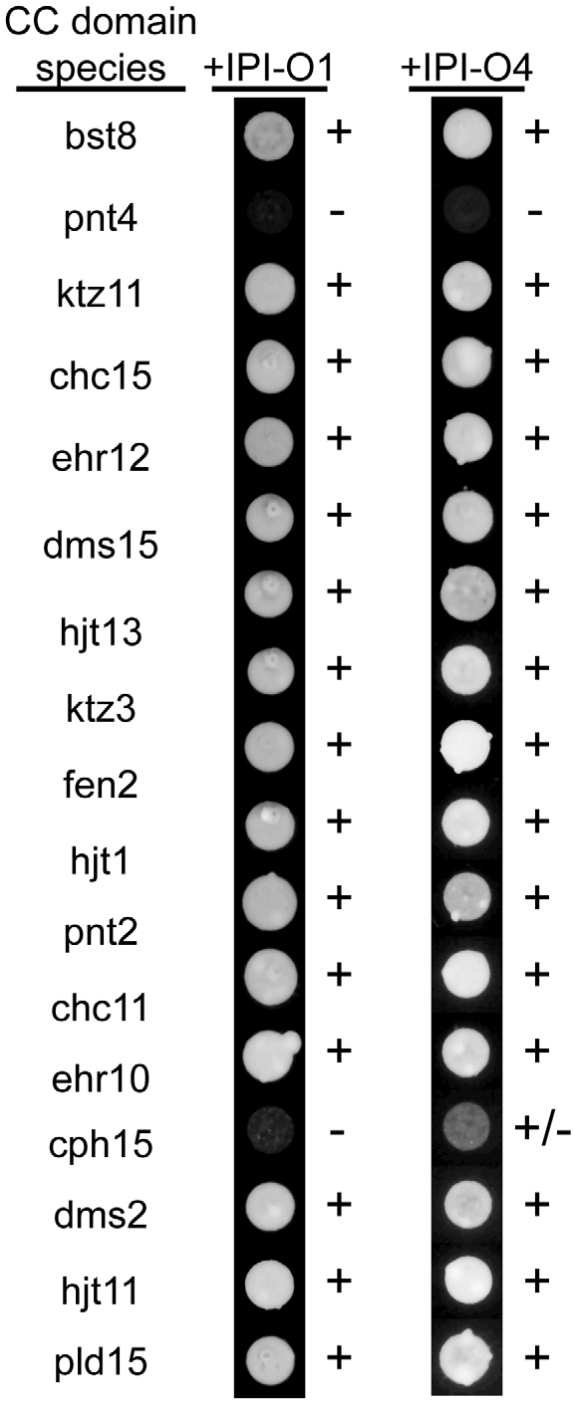
Interactions between RB-like CC domains from potato species with either IPI-O1 or IPI-O4. The three-letter abbreviation for each species is shown. Numbers after the species name represents the PCR clone number. + and − signs to the right of the yeast colonies indicate positive or negative interaction, respectively. Photos were taken after 8 days of growth on selective media. Photos represent the results of three independent yeast transformations.

The interaction between IPI-O4 and CC domains of RB-like sequences from ten wild species of potato was tested. These ten species were not chosen based on their late blight resistance phenotype but rather because they capture a large amount of diversity within a small number of species. Seventeen different CC domains from these species, sharing deduced amino acid sequence similarities ranging between 65.9% and 98.2% (Figs. S4, S5), were chosen for interaction testing with IPI-O1 and IPI-O4. Fifteen of the 17 RB-like CC fragments interacted with both IPI-O1 and IPI-O4 in yeast (Figure 2). Only one fragment from *S. cardiophyllum* showed a similar interaction pattern as the *S. bulbocastanum* RB CC domain in yeast. It did not interact with IPI-O1, but we did observe a weak but consistent interaction with IPI-O4. This weak interaction was characterized by slower growth of the yeast during selection. One fragment from *S. pinnatisectum* showed no interaction with either IPI-O1 or IPI-O4. Protein blotting showed that the pnt4 and cph15 CC domains in the pSOS expression vector were stable in yeast (Figure S2).

### Specific mutations within IPI-O4 abolish interaction with the RB CC domain

**Figure 3:**
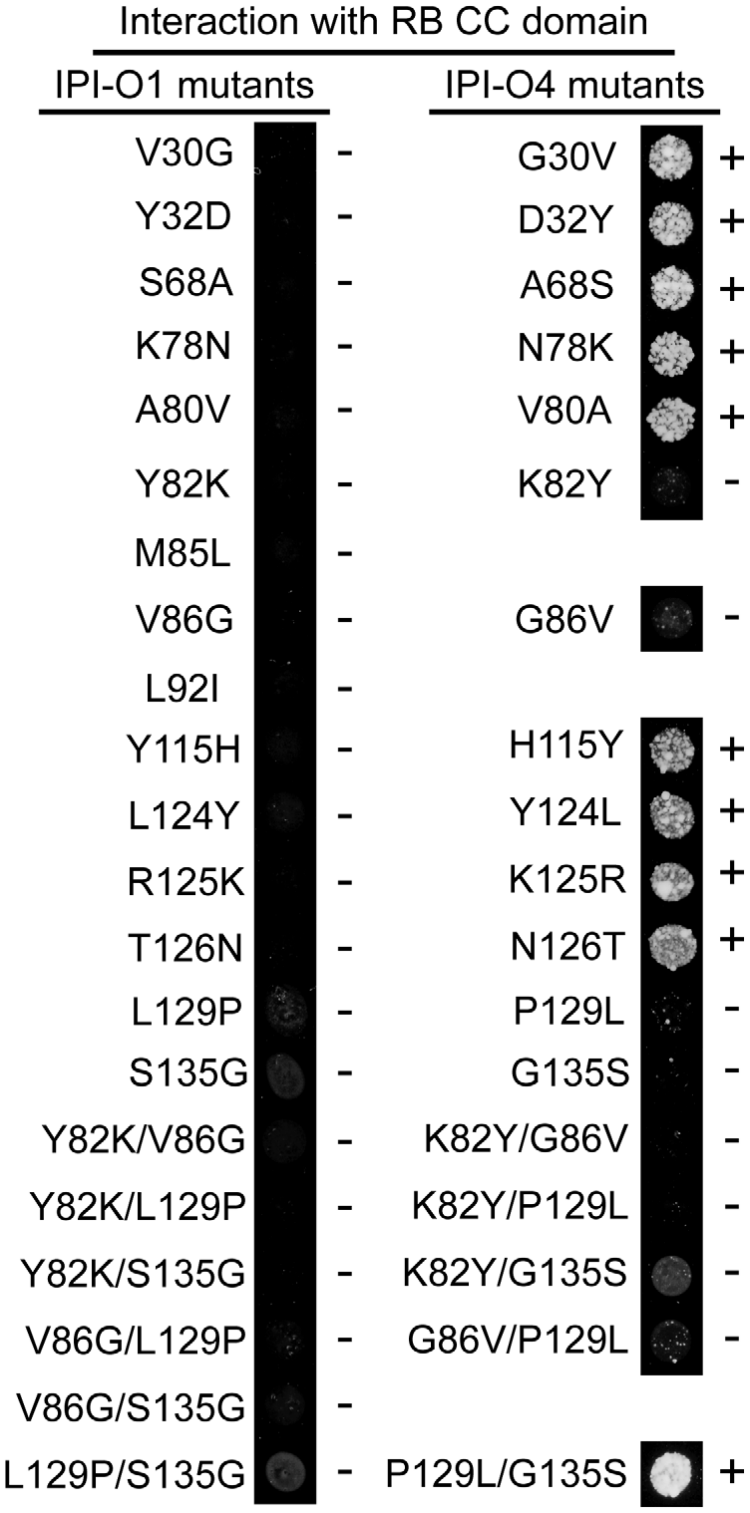
Yeast two-hybrid interactions between the CC domain of RB and IPI-O1/4 single and double amino acid mutants. + and − signs to the right of the yeast colonies indicate positive or negative interaction, respectively. Pictures were taken after 8 days of growth on selective media. All the experiments were performed three times.

Twenty amino acids differentiate IPI-O1 from IPI-O4 [30]. In order to determine which residue(s) plays a role in the RB CC/IPI-O4 interaction, individual IPI-O1 and IPI-O4 amino acids were mutated to the corresponding residue in the other effector. Fifteen IPI-O1 mutants and 13 IPI-O4 mutants with single amino acid changes were tested for interaction with the RB CC domain. Despite extensive efforts, we could not obtain mutations at the other locations. None of the 15 IPI-O1 single amino acid mutants interacted with the CC domain (Figure 3). However, four IPI-O4 mutations, K82Y, G86V, P129L and G135S abolished or weakened the RB CC domain/IPI-O4 interaction (Figure 3). In order to determine whether variant amino acids at these four sites function together to alter the IPI-O/CC interaction phenotype, 6 IPI-O1 double amino acid mutants, and 5 IPI-O4 double amino acid mutants were tested (Figure 3). The results showed that none of the IPI-O1 mutants interacted with the CC domain. One IPI-O4 double amino acid mutant, IPI-O4 P129L/G135S, regained its interaction with the RB CC domain. Protein blotting showed that all of the IPI-O single and double mutants in the pSOS expression vector were stable in yeast (Figure S2).

### Amino acid 129 of IPI-O1 is critical for RB HR elicitation

**Figure 4:**
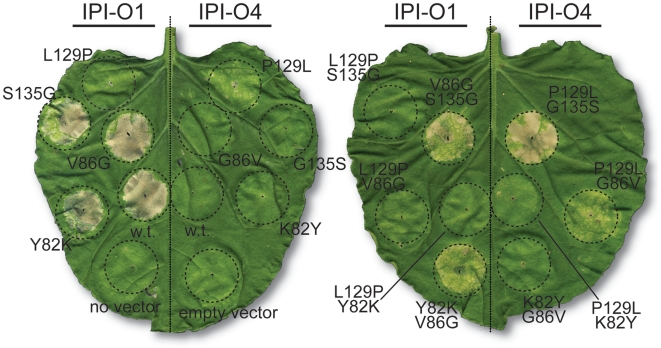
Induction of hypersensitive cell death by IPI-O1 and IPI-O4 mutants *in planta*. *A. tumefaciens* strains expressing IPI-O, IPI-O mutants, or the indicated controls were infiltrated into leaves of *RB* transgenic *N. benthamiana* plants. Leaves were photographed at 6 days after infiltration.

The four mutations at amino acids 82, 86, 129, and 135 of IPI-O1 and IPI-O4 were assayed for an effect of activation of HR elicitation by agroinfiltration in *RB*-transgenic *N. benthamiana*. These four amino acids were chosen because they significantly impacted the interaction between the RB CC domain and IPI-O4. The mutant IPI-O1 L129P failed to elicit the HR, while the other single amino acid mutants were still capable of inducing the HR (Figure 4). Consistent with these results, IPI-O1 double mutants containing L129P, namely IPI-O1 Y82K/L129P, V86G/L129P, and L129P/S135G, also lost the ability to elicit an RB-mediated HR. The two IPI-O1 mutants Y82K/V86G and V86G/S135G induced an HR, demonstrating that they were still recognized by RB. When present, the cell death response began approximately 48 hours after infiltration (hai). No visible differences were observed regarding timing of the onset of the HR or the cell death intensity between wild type IPI-O1 and the mutants. Protein blotting indicated that IPI-O1 mutants containing L129P were present at detectable amounts and stable in *N. benthamiana* leaves (Figure S6).

The HR induction phenotype of IPI-O4 mutants also supports the conclusion that L129P is important in RB activation. All P129L-containing mutants of IPI-O4 (P129L, K82Y/P129L, G86V/P129L and P129L/G135S) gained the ability to elicit the HR in *N. benthamiana* with *RB* (Figure 4). In contrast, mutants of IPI-O4 K82Y, G86V, G135S and K82Y/G86V did not elicit the HR.

### Effect of mutations on suppression of RB HR induction

**Figure 5:**
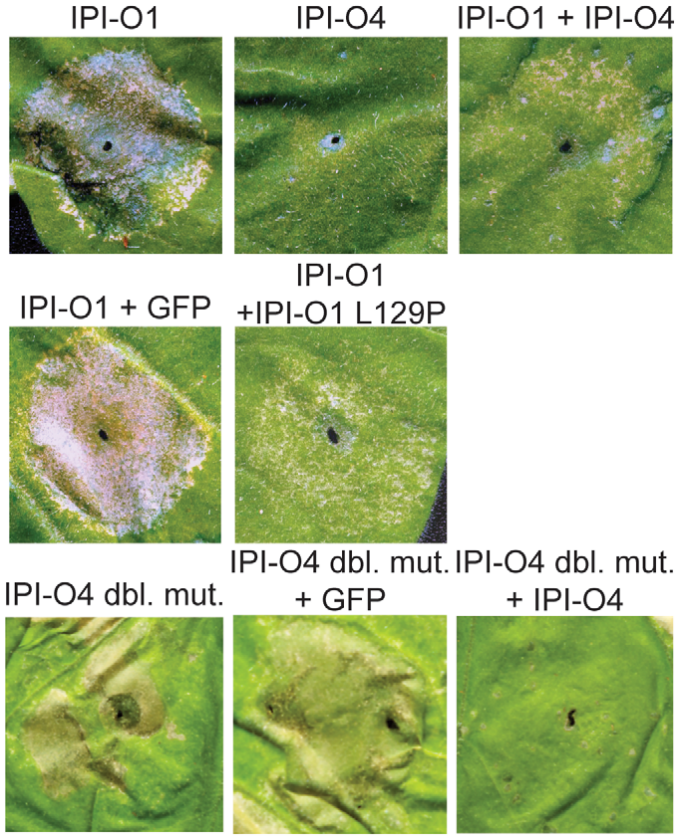
Suppression of hypersensitive cell death of IPI-O1 by IPI-O1 L129P and IPI-O4 P129L/G135S (double mutant) by IPI-O4. *A. tumefaciens* strains expressing the indicated IPI-O variants or a GFP control were infiltrated or co-infiltrated into leaves of RB transgenic *N. benthamiana* plants. Leaves were photographed at 6 days after infiltration.

We coinfiltrated *RB*-transgenic *N. benthamiana* leaves with IPI-O1 and IPI-O1 L129P to test whether this mutant is able to suppress RB-mediated HR elicitation (Figure 5; Figure S7). Compared to IPI-O1 alone or coinfiltration of IPI-O1 with GFP, no HR or faint HR was observed in areas co-infiltrated with IPI-O1 and IPI-O1 L129P, indicating a suppression of cell death by IPI-O1 L129P. However, cell death was not suppressed when INF1 and IPI-O1 L129P were coexpressed (Figure S7), demonstrating that IPI-O1 L129P suppression of cell death is RB-specific.

As noted previously, the double mutant IPI-O4 P129L/G135S is able to induce the HR in the presence of RB. We tested the ability of IPI-O4 to suppress this HR response by co-infiltrating *RB*-transgenic *N. benthamiana* leaves with these two effectors (Figure 5). HR induced in RB transgenic leaves by IPI-O4 P129L/G135S alone was observed 48 hai. Similar to regions exposed to GFP or IPI-O4 alone (Figure S8), no HR or faint HR was observed in areas co-infiltrated with IPI-O4 P129L/G135S and IPI-O4, indicating a suppression of cell death in the presence of IPI-O4 (Figure 5). Coexpression of GFP with IPI-O1 or IPI-O4 P129L/G135S did not abrogate HR elicitation, nor did coexpression of IPI-O4 and INF1, demonstrating specificity for RB. We then tested the effect that single amino acid changes within IPI-O4 had on its ability to suppress the HR. Coexpression of the IPI-O4 single amino acid mutants K82Y, G86V, and G135S with IPI-O1 revealed an effect on HR suppression, although their suppressive activity appeared to be lower than IPI-O4 or the IPI-O1 L129P mutant (Figure S9). IPI-O4 P129L was not tested because it is able to elicit the HR itself.

## Discussion

The interaction between R proteins and pathogen effectors determines the resistance or susceptibility phenotype of the host. *P. infestans* has repeatedly proven itself capable of overcoming major resistance genes. Although many resistance genes have been identified in wild potato species and have been integrated into new potato varieties, this has typically provided only temporary late blight control. Our ability to understand how these resistance genes are able to recognize pathogen effectors and elucidate the mechanisms that allow pathogens to overcome resistance will assist us in predicting the ability of *P. infestans* to overcome resistance and permit engineering or identifying genes that can resist suppression by specific effectors. The *P. infestans* effectors IPI-O1 and IPI-O4 provide opportunities to study the complex molecular mechanisms of R protein-effector interactions, since IPI-O1 elicits RB-mediated resistance while IPI-O4 functions to suppress resistance elicitation.

In this study, self-association of the RB CC domain was detected, suggesting that RB may dimerize or polymerize through this domain. Dimerization or polymerization has been reported in different types of R proteins after they are activated upon recognition of their cognate effectors. The tobacco N protein oligomerizes in the presence of the effector p50 [9]. N protein oligomerization correlates with resistance, since mutations in the TIR domain that abolish self-association also abolish resistance [9]. The CC domain of Arabidopsis R protein RPS5 also forms oligomers [11], but it is currently unknown whether interaction with the AvrPphB effector or the PBS1 accessory protein affects R protein oligomerization. The tomato Prf protein forms oligomers through self-association of the N-terminus [35]. This region of the protein is also important in interactions with important cofactors, such as Pto, Fen, Pth3, and Pth5 [35]. The barley MLA protein also self-associates through interaction of its CC domain [36], but unlike RB, MLA effector recognition specificity lies within the LRR and not the CC domain [37]. Our results indicating oligomerization of RB are based on protein-protein interaction tests in yeast and *in planta* using only the CC domain and, without data using the full-length protein we can only hypothesize that RB oligomerization is necessary for proper activity. We have provided additional evidence that this region of RB is important in HR elicitation through its interaction with IPI-O effectors. Since R protein oligomerization is an important function in similar resistance responses, it is not unexpected that suppression of this event would be a logical target for a pathogen effector. We observed an interaction between IPI-O4 and the CC domain of RB in yeast and *in planta* and between IPI-O1 and the CC domain *in planta*, suggesting that IPI-O may affect RB function through interaction with this region, thereby preventing CC oligomerization or blocking an interaction with other signaling components. In addition, we observed an interaction between most RB-like CC fragments from wild species of potato and IPI-O1, as well as with IPI-O4. This observation suggests that IPI-O effectors may have defeated ancient RB alleles by preventing CC oligomerization or blocking an interaction with other signaling components.

Effectors from different types of pathogens have been found to suppress host basal defense or hypersensitive cell death induced by elicitors or effectors even in the presence of cognate R proteins [38]. The Avr1 effector from *Fusarium oxysporum* f.sp. *lycopersici* (Fol) suppresses resistance mediated by the tomato *I-2* and *I-3* genes, but tomato plants with either *I* or *I-1* are able to recognize Avr1 to elicit resistance [39]. The tomato protein kinase Pto confers resistance to *P. syringae* expressing AvrPtoB, a ubiquitin ligase [40], [41]. Despite 80% amino acid similarity to Pto, the Fen kinase is not able to elicit resistance in the presence of AvrPtoB. This is due to suppression of Fen through ubiquitination and degradation by AvrPtoB [42]. Pto phosphorylates AvrPtoB to inactivate its E3 ligase activity and mediate ETI together with Prf [43], [44]. We have previously shown that IPI-O4 is capable of inhibiting the HR elicited by IPI-O1 in *N. benthamiana* expressing *RB* [25]. In the present study, no physical interaction between IPI-O1 and IPI-O4 was detected, suggesting that IPI-O4 does not inhibit IPI-O1 recognition by interacting with IPI-O1 directly. However, we cannot rule out that conditional post-translational modifications or the environment *in planta* might result in a direct interaction. The ability of the IPI-O1 L129P mutant to suppress IPI-O1 elicitation of the HR in the presence of RB demonstrates a critical step in the evolution of this effector family. This finding suggests that a simple mutation at this location in IPI-O1 could alter pathogen virulence when RB is present. Our previous analysis of IPI-O variants from *P. infestans* isolates identified two alleles outside of the IPI-O4 class that contain a proline at amino acid 129 [25]. The first, CMPh0-07.04 derived from a Thai isolate, is unique but falls within the IPI-O3 family. The second, 68.12 from a Guatemalan isolate, is likely the result of a recombination event between IPI-O4 and another IPI-O family member. The virulence of isolate CMPh0-07 was not tested, but isolate 68 showed increased aggressiveness on plants with and without the *RB* gene indicating its ability to suppress resistance responses [25].

The utilization of transient effector expression in *N. benthamiana* has allowed us to rapidly identify specific amino acids within a pathogen effector that condition host resistance. Four amino acids in IPI-O play an important role in RB CC/IPI-O4 interaction and one of them also determines IPI-O1 recognition by RB. All four amino acids are located C-terminal to the RXLR motif, which is consistent with the fact that the RXLR motif is responsible for translocation [33], [34], while the C-terminal half conditions virulence and avirulence [24], [45]. Amino acid 129 is within the W motif and 135 is adjacent to the W motif [24], [25]. The conformational rigidity of proline and the relative flexibility of glycine suggest that they might have an important role in structural changes that alter the presentation of loops within the IPI-O4 protein. The IPI-O amino acid sequence shares no similarity to any known protein molecules and no definitive function within the host cytoplasm has been determined. The IPI-O mutants obtained in this study will help to predict the structure and properties of IPI-O, and additionally, the crystal structure of IPI-O in the presence of the RB CC domain would help to complete elucidation of the molecular mechanisms surrounding the IPI-O/RB interaction.

The two single amino acid mutants of IPI-O4, P129L and G135S, lost the ability to interact with the RB CC domain. However, the double mutant IPI-O4 P129L/G135S elicited the HR and regained the ability to interact with the CC domain, demonstrating a key link between IPI-O/CC interaction and resistance elicitation. These results also suggest that HR elicitation by IPI-O4 P129L/G135S is epistatic to interaction with the CC domain. In other words, interaction with the CC domain is not sufficient to inhibit the HR *in planta* if the effector molecule itself is recognized by RB and triggers the HR. The important role of CC domain interaction is further demonstrated by the fact that IPI-O4 mutants that are compromised in RB CC domain interactions are also compromised in suppressing HR induced by IPI-O1.

**Figure 6:**
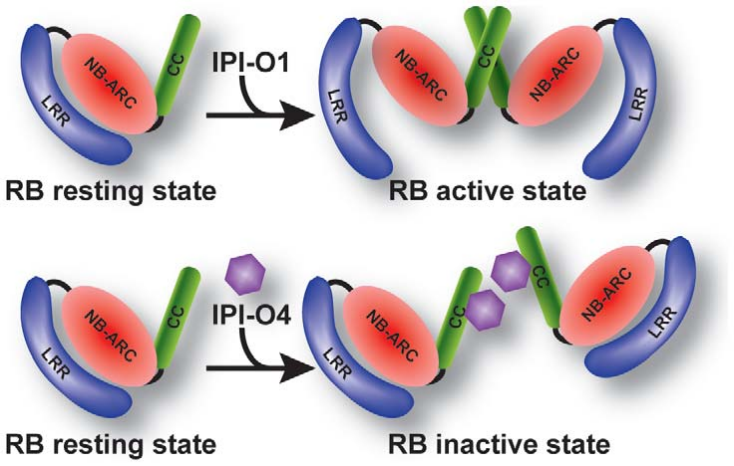
Model for RB/IPI-O interactions. The top panel represents a resistance response. In the absence of IPI-O1, RB remains in a resting state. The presence of IPI-O1 elicits a conformational change that enables RB oligomerization through the CC domain and leads to an activated protein state. As shown in the bottom panel, when IPI-O4 is present, this effector interacts with the CC domain and prevents CC oligomerization, thus suppressing RB activation.

Collectively, our data, combined with data from other CC-NB-LRR proteins, is consistent with the model shown in Figure 6. The model suggests that in the absence of IPI-O, RB remains in a resting state. CCNB stabilizes this state. A conformational change occurs upon recognition of IPI-O1, which enables RB to oligomerize through the CC domain and expose a platform for signaling components or leads to an activated protein state. However, when IPI-O4 is present, this effector interacts with the CC domain and prevents CC oligomerization, or blocks an interaction with other signaling components, thus suppressing RB activation. Our data indicates that IPI-O1 also interacts with the RB CC domain *in planta*, but not in yeast. The IPI-O1 fusion protein is stable in yeast, suggesting that perhaps an additional accessory protein, found only *in planta*, is necessary for stability of the interaction with the RB CC domain. Our simplified model does not account for this. Combined with the fact that GFP:IPI-O1 fusion protein was not as abundant as GFP:IPI-O4 in *N. benthamiana* leaves, we hypothesize that IPI-O1 protein accumulation is affected by factors in the plant cell that interfere with its ability to suppress RB oligomerization and elicit resistance.

Our data demonstrates a co-evolutionary arms race between IPI-O and RB. The fact that several late blight susceptible potato species contain RB-like proteins with CC domains that interact with both IPI-O1 and IPI-O4, suggests that *P. infestans* delivery of IPI-O effectors into the plant cell could be responsible for the pathogen's ability to elude recognition and cause disease. If this is true, *S. bulbocastanum* RB is one of the latest in a line of R proteins that are able to recognize the presence of IPI-O1 while escaping suppression by this effector. Our ability to understand the molecular interactions that condition resistance and suppression will hopefully allow us to design or identify RB alleles that can continue to effectively control disease epidemics. A *RB* homolog that recognizes IPI-O4 has been found in the wild species of potato *S. stoloniferum* [26] demonstrating that variants of the R protein exist that are able to avoid suppression by IPI-O4. In our study, a CC homolog from *S. pinnatisectum* showed no interaction with either IPI-O1 or IPI-O4. We are currently pursuing research focused on answering whether an engineered RB containing the CC domain from *S. pinnatisectum* may also escape suppression by IPI-O4. Despite the fact that *P. infestans* isolates that overcome *RB* resistance have been found [24], [25], *RB* remains valuable for potato breeding due to the almost ubiquitous existence of IPI-O1 in strains from major potato growing regions. However, due to the adaptive nature *P. infestans* and the continual proliferation of strains of *P. infestans*, we expect that the benefits of using the *RB* gene for late blight resistance will be ephemeral. Significant efforts must continue to be made to identify and integrate novel resistance. Pyramiding of *RB*, *RB* orthologs, and modified *RB* recognizing different IPI-O variants is one strategy that could be used to expand the durability of this gene.

## Materials and Methods

### Yeast two-hybrid assays

The CytoTrap system (Agilent) was used to detect protein-protein interactions. IPI-O1, IPI-O4, full length RB [amino acids (aa) 1–970], the CC domain of RB (aa 1–165), the NB domain of RB (aa 166–521), the CC-NB domain of RB (aa 1–521) and the LRR domain of RB (aa 522–970) were amplified with primers listed in Table S1 using Platinum PCR SuperMix High Fidelity (Invitrogen). IPI-O1 (PexRD6-1) and IPI-O4 (PexRD6-3) targets were those described in Vleeshouwers et al. [26]. The PCR products were ligated into *BamH*I and *Sal*I digested pSos or *EcoR*I and *Sal*I digested pMyr, respectively.

### Yeast protein extraction and blotting

Cdc25 yeast strains containing RB domains or IPI-O variants in pSos were grown to OD_600_ = 0.6 at 24°C in 10 ml SD/glucose (-L) media. Yeast cells with no pSos vector were grown in YPAD broth. Protein purification was performed according to the CytoTrap system instruction manual. Protein pellets were resuspended in 300 µl of SU buffer. 20 µl of each protein sample was heated at 65°C for 3 minutes, separated by 10% SDS-PAGE and transferred to Hybond-P (GE Healthcare). Immunoblotting was performed using a mouse monoclonal anti-Sos primary antibody (1∶250, BD Biosciences) and an anti-mouse secondary antibody produced in goat (1∶10000, Sigma). Antibody detection was carried out using an ECL Western blotting detection system (GE Healthcare) according to the manufacturer's instructions.

### Cloning of RB-like CC domains from wild potato

Seeds from 10 wild species of potato, shown in Table 1, were obtained from the National Research Support Program (NRSP)-6 potato GeneBank in Sturgeon Bay, Wisconsin. Seedlings were grown under greenhouse conditions (23°C day/15°C night temperatures with 14 hours of light) and watered as needed. Total RNA was extracted from young leaves using a Total RNA Extraction Kit (Sigma) according the manufacturer's instructions. One µg of DNase-treated RNA was reverse transcribed using a First-strand Synthesis Kit (Bio-Rad). cDNA was used as a template to amplify the *RB* homologous CC fragments using primers RBFORBAMHI and RBCCREVSALI (Table S1). PCR conditions and cloning into pSos vectors was carried out as described above.

**Table 1:** Wild potato species used for amplification of *RB* homologous CC fragments.

Species	Abbreviation	PI number
*Solanum brachistotrichum*	bst	279244
*Solanum chacoense*	chc	275138
*Solanum cardiophyllum*	cph	283062
*Solanum demissum*	dms	161366
*Solanum ehrenbergii*	ehr	184762
*Solanum fendleri*	fen	225661
*Solanum hjertingii*	hjt	283103
*Solanum kurtzianum*	ktz	472923
*Solanum polyadenium*	pld	320342
*Solanum pinnatisectum*	pnt	186553

### Site-directed mutagenesis

Mutants of *P. infestans* effectors IPI-O1 and IPI-O4 were generated by circular PCR using IPI-O1 and IpiO4 in pMyr as templates. A pair of oligonucleotide primers containing the desired nucleotide substitution (Table S1), each complementary to the opposite strands of the same target sequence, were extended during temperature cycling using *PfuTurbo* DNA polymerase (Agilent). After the temperature cycling, the PCR products were treated with *Dpn*I for 2 hr at 37°C to digest the methylated parental DNA template and then transformed into *E. coli*. The resultant mutated plasmid constructs were verified by sequencing.

### HR induction assays in *N. benthamiana*

The wild type and mutant IPI-O1 and IPI-O4 in pMyr were used as templates for PCR amplification to incorporate *BamH*I and *Sac*I restriction enzyme sites. Primer sequences are listed in Table S1. PCR products were ligated into *BamH*I and *Sac*I digested binary vector pBI121. All constructs were introduced into *A. tumefaciens* strain GV3101 by electroporation.

Wild type and *RB*-transgenic *N. benthamiana* plants [46] were grown in pots in a walk-in growth chamber with 28°C day/27°C night temperatures, 16 hours of light, and were watered and fertilized as needed. Four to six weeks old plants were used for agroinfiltration. Agroinfiltration was carried out as previously described [26]. Infiltrated plants were maintained in a walk-in growth chamber (22°C day/18°C night temperatures with 16 h of light). When testing the HR suppression, HR inducing constructs (INF1, IPI-O1, IPI-O4 P129L/G135S) were infiltrated at the same infiltration spot 24 hours after the infiltration of non-HR inducing constructs (GFP, IPI-O4, IPI-O1 L129P, IPI-O4 K82Y, IPI-O4 G86V, IPI-O4 G135S).

### Protein extraction, blotting and co-immunoprecipitation

The wild type and mutant IPI-O1, IPI-O4 and the RB CC domain in pMyr were used as templates for PCR amplification to incorporate *BamH*I and *Xho*I restriction enzyme sites. Primer sequences are listed in Table S1. PCR products were ligated into *BamH*I and *Xho*I digested pENTR1a (Invitrogen). Gateway LR reactions (Invitrogen) were performed to introduce wild type IPI-O1 and mutants into the binary vector pEG201 [47] with an N-terminal HA tag, and introduce IPI-O1, IPI-O4, and the RB CC domain into pGWB6 and pGWB18 [48] with N-terminal GFP and Myc tags respectively. Infiltrated areas of *N. benthamiana* leaves were harvested 21 hours after agroinfiltration. To prepare total protein, plant material was ground in liquid nitrogen and thawed in extraction buffer containing 50 mM Tris, pH 7.5, 150 mM NaCl, 0.1% Triton X-100, and a protease inhibitor cocktail according to the manufacturer's instructions (Sigma). Samples were incubated on ice for 1 hour. The suspension was centrifuged at 4000 g at 4°C 6 times, each time for 10 min. In the protein blot assay, 100 mg of ground plant material was thawed in 100 µl of extraction buffer, and 25 µl of the supernatant was resolved on a 12% SDS-PAGE gel and transferred to Hybond-P (GE Healthcare) according to the manufacturers recommendations. Protein was detected using an anti-HA-peroxidase antibody (Roche) with an ECL Western blotting detection system (GE Healthcare) according the manufacturer's instructions. In the co-immnunoprecipitation assay, 1 gram of ground plant material was thawed in 1 ml of extraction buffer. Seven µl of anti-GFP antibody (Clontech) was added to each protein sample, followed by incubation for 1 hour with end-to-end shaking at 4°C. 50 µl of pre-equlibrated protein A agarose (Roche) was then added to each sample and incubated for 4 hours with end-to-end shaking at 4°C. The beads were spun at 4000 g at 4°C and washed with 1 ml of wash buffer (50 mM Tris (pH 7.5), 150 mM NaCl) 6 times (15 min each time) with end-to-end shaking at 4°C. After washing, the beads were eluted with 45 µl of 2× SDS loading buffer and heated at 95°C for 7 min. The eluted protein was separated on a 10% SDS-PAGE gel and transferred to PVDF membrane and detected with anti-Myc-HRP antibodies (Sigma) using an ECL Western blotting detection system (GE Healthcare).

## Supporting Information

Figure S1 Yeast two-hybrid screening of RB domain interactions. Each panel shows three independent transformants of identical genotypes. Positive and negative controls were provided by the CytoTrap system manufacturer. RB = full-length RB; CC = RB coiled-coil domain; NB = RB nucleotide binding domain; LRR = RB leucine-rich repeat domain. All spots contained similar quantities of yeast at the time of plating. Pictures were taken after 8 days of growth on selective media.
(TIF)

Figure S2 Protein blotting showing stability of IPI-O and CC SOS-domain fusions in yeast. A–D) Total yeast proteins were separated on separate acrylamide gels, blotted, and probed with SOS-specific antibody. Ponceau S stained PVDF membranes are shown below the results of protein blotting and antibody detection.
(TIF)

Figure S3 Protein blotting of the input protein for co-immunoprecipitation. Total protein from leaf sections agroinfiltrated with the indicated constructs was extracted and separated on an acrylamide gel. Protein was blotted and detected using a GFP-specific antibody (A) or a Myc-tag specific antibody (B). Ponceau S stained PVDF membranes are shown to demonstrate equal loading.
(TIF)

Figure S4 Amino acid sequence identity chart showing the pairwise percent identity between RB CC domains amplified from wild potato species. Sequence names contain the three-letter abbreviation for each species. Numbers after the species name represents the PCR clone number.
(TIF)

Figure S5 Sequence alignment of RB CC domain deduced amino acid sequences. Letters in black boxes are identical to the consensus sequence (shown at top of each row). Sequence names contain the three-letter abbreviation for each species. Numbers after the species name represents the PCR clone number.
(TIF)

Figure S6 IPI-O1 mutants containing L129P are stable in *RB*-transgenic *N. benthamiana* leaves. A protein blot was performed using total protein extracts following agroinfiltration with constructs expressing indicated HA-IPI-O1 mutants. The 18-kDa protein band represents the expected size of recombinant IPI-O1 mutants. Lane1: wild type IPI-O1; Lane2: non-infiltrated *N. benthamiana*; Lane3: IPI-O1 L129P; Lane4: IPI-O1 Y82K/L129P; Lane5: IPI-O1 V86G/L129P; Lane6: IPI-O1 L129P/S135G.
(TIF)

Figure S7 IPI-O1 L129P inhibits the HR induced by IPI-O1. *A. tumefaciens* strains expressing IPI-O mutants or the indicated controls were infiltrated into leaves of *RB* transgenic *N. benthamiana* plants. Leaves were photographed at 6 days after infiltration.
(TIF)

Figure S8 IPI-O4 inhibits the HR induced by the IPI-O4 P129L/G135S double mutant. *A. tumefaciens* strains expressing IPI-O mutants or the indicated controls were infiltrated into leaves of *RB* transgenic *N. benthamiana* plants. Leaves were photographed at 6 days after infiltration.
(TIF)

Figure S9 IPI-O4 K82Y, G86V, and G135S inhibit the HR induced by IPI-O1. *A. tumefaciens* strains expressing IPI-O mutants or the indicated controls were infiltrated into leaves of *RB* transgenic *N. benthamiana* plants. Leaves were photographed at 6 days after infiltration. Note that the inhibitory effect of these mutants is not as strong as that of IPI-O1 L129P since some cell death was still observed in the area coinfiltrated with IPI-O1 and IPI-O4 K82Y (A), G86V (B), or G135S (C).
(TIF)
