# Correction: Molecular Determinants of Resistance Activation and Suppression by *Phytophthora infestans* Effector IPI-O

**DOI:** 10.1371/annotation/75775518-f06e-4148-a639-31cfc6972b2e

**Published:** 2012-09-11

**Authors:** Yu Chen, Zhenyu Liu, Dennis A. Halterman

Table S1 does not appear. Please view Table S1 here: 
