# Supplementary figures and images for: Molecular Determinants of Resistance Activation and Suppression by Phytophthora infestans Effector IPI-O
Source: PLoS Pathog. 2012 Mar 15;8(3):e1002595. doi: 10.1371/journal.ppat.1002595 (PMC3305431; doi:10.1371/journal.ppat.1002595)

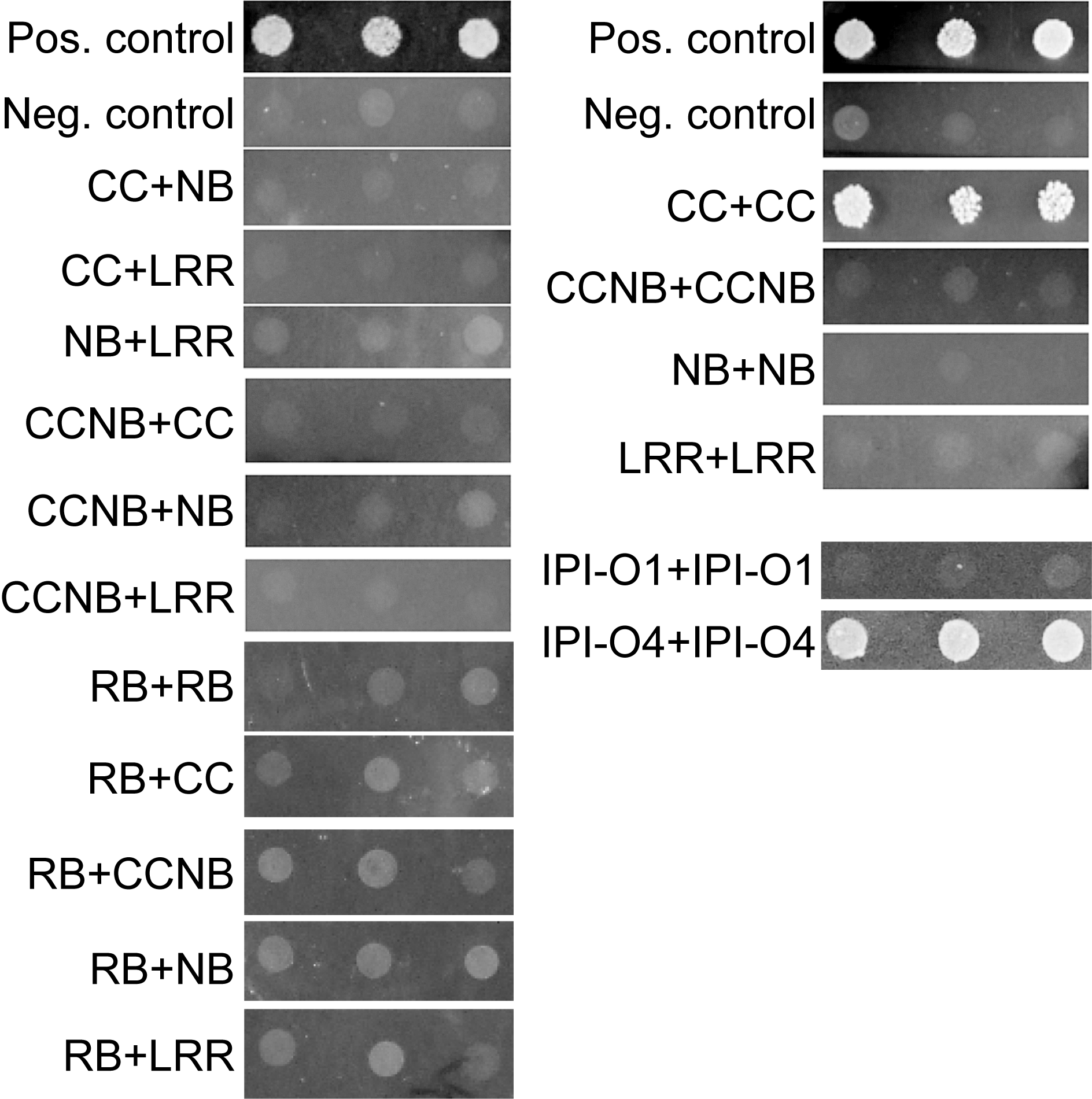

Supplement: Figure S1 — Yeast two-hybrid screening of RB domain interactions. Each panel shows three independent transformants of identical genotypes. Positive and negative controls were provided by the CytoTrap system manufacturer. RB = full-length RB; CC = RB coiled-coil domain; NB = RB nucleotide binding domain; LRR = RB leucine-rich repeat domain. All spots contained similar quantities of yeast at the time of plating. Pictures were taken after 8 days of growth on selective media. (TIF) [file ppat.1002595.s001.tif]

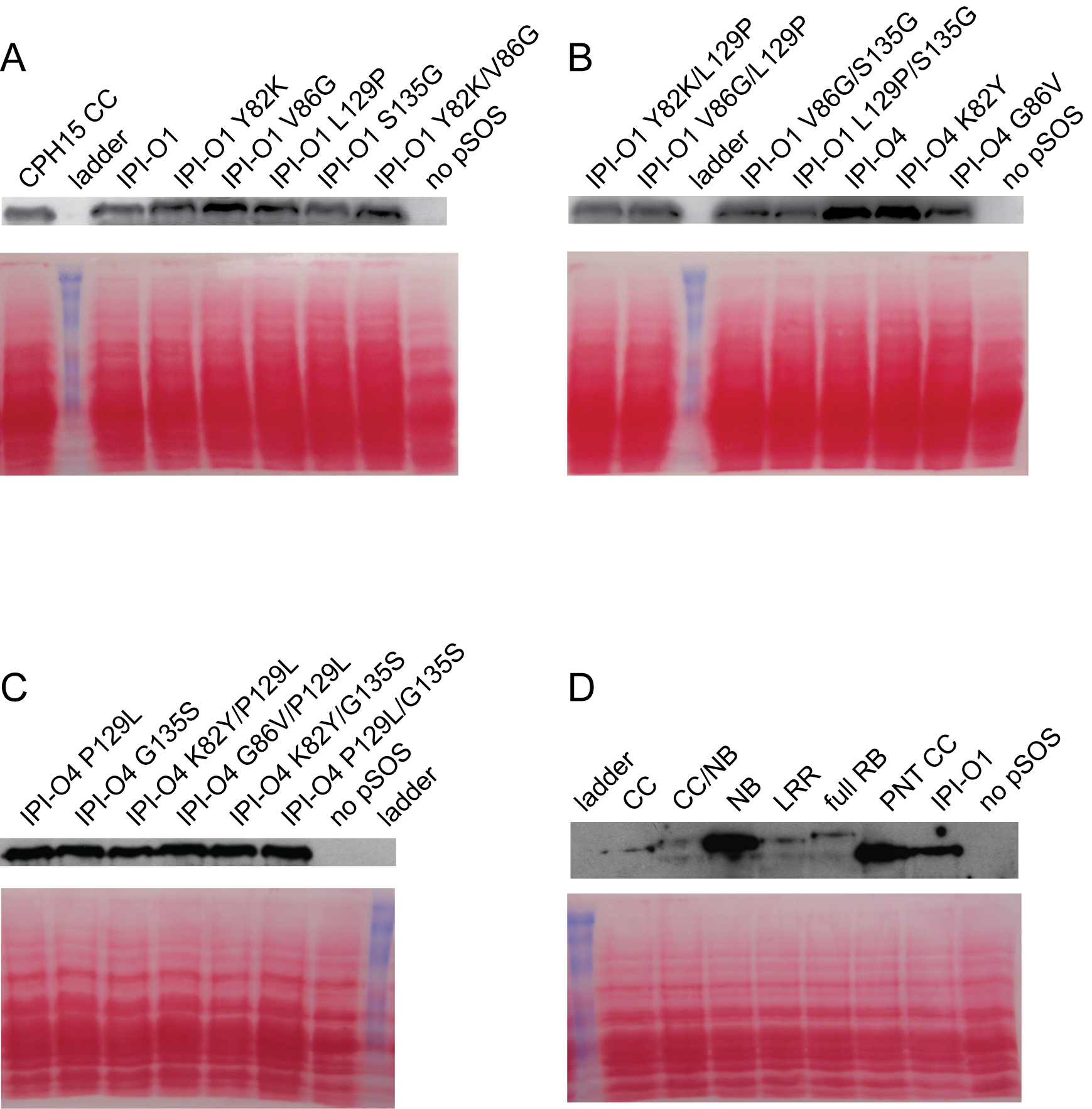

Supplement: Figure S2 — Protein blotting showing stability of IPI-O and CC SOS-domain fusions in yeast. A–D) Total yeast proteins were separated on separate acrylamide gels, blotted, and probed with SOS-specific antibody. Ponceau S stained PVDF membranes are shown below the results of protein blotting and antibody detection. (TIF) [file ppat.1002595.s002.tif]

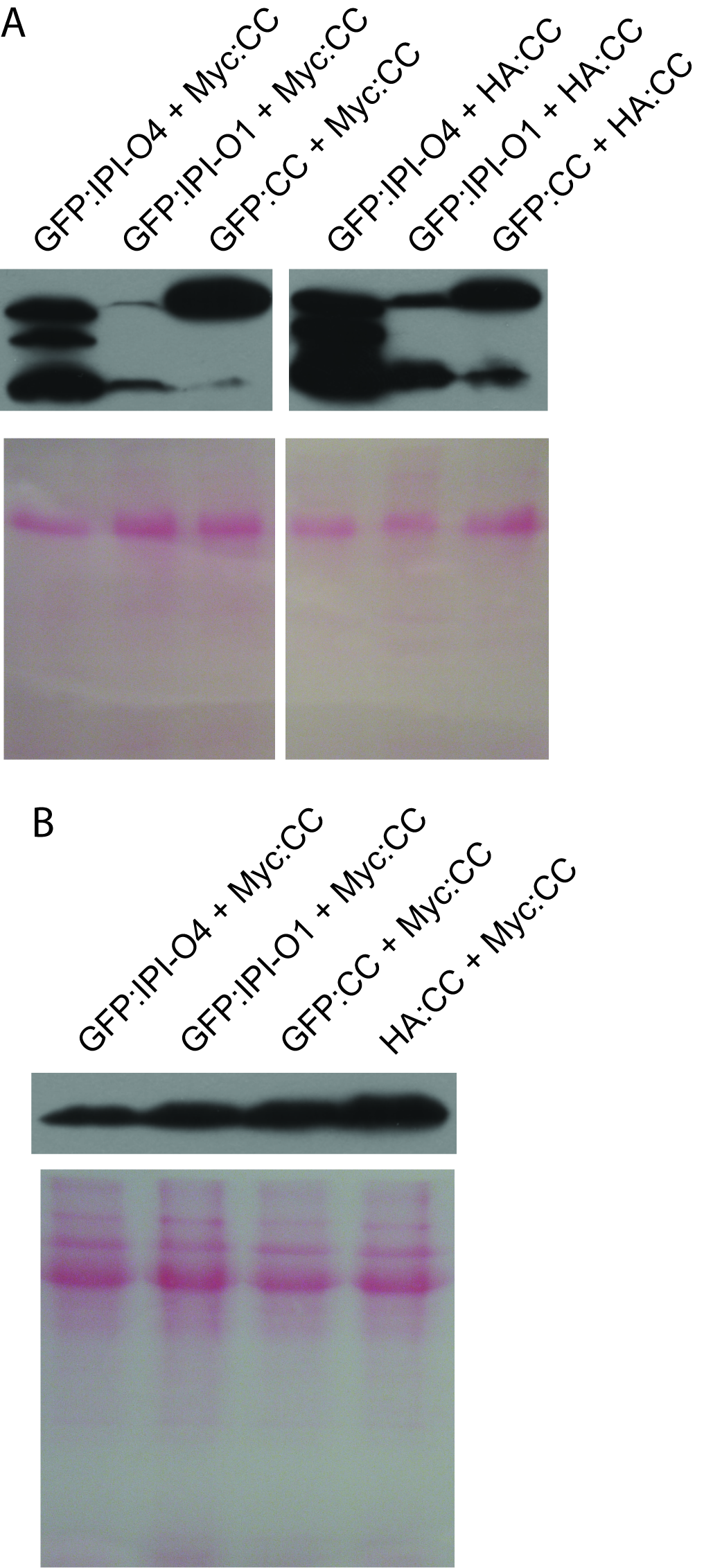

Supplement: Figure S3 — Protein blotting of the input protein for co-immunoprecipitation. Total protein from leaf sections agroinfiltrated with the indicated constructs was extracted and separated on an acrylamide gel. Protein was blotted and detected using a GFP-specific antibody (A) or a Myc-tag specific antibody (B). Ponceau S stained PVDF membranes are shown to demonstrate equal loading. (TIF) [file ppat.1002595.s003.tif]

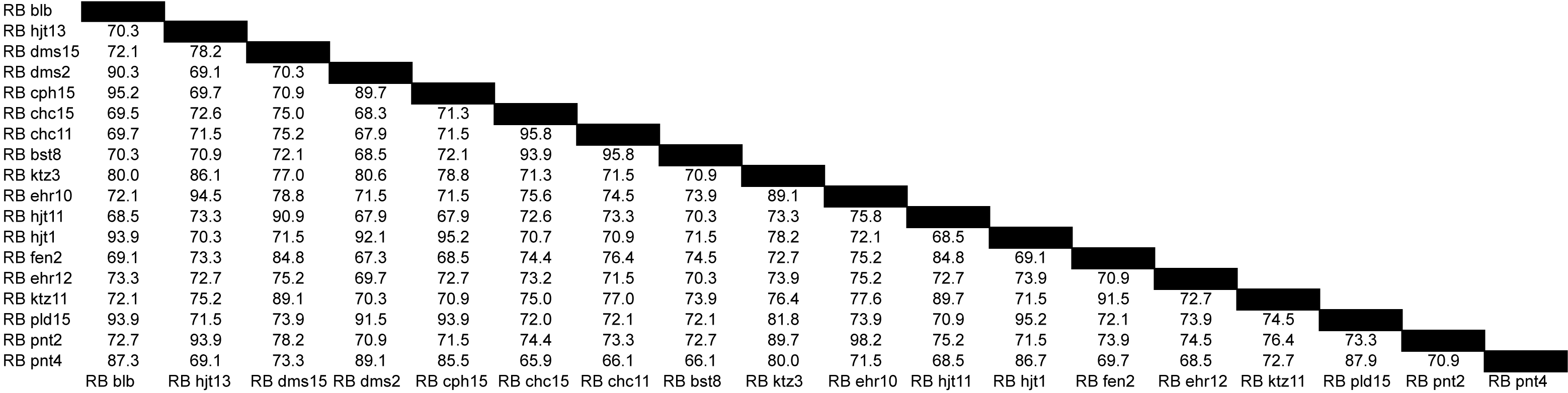

Supplement: Figure S4 — Amino acid sequence identity chart showing the pairwise percent identity between RB CC domains amplified from wild potato species. Sequence names contain the three-letter abbreviation for each species. Numbers after the species name represents the PCR clone number. (TIF) [file ppat.1002595.s004.tif]

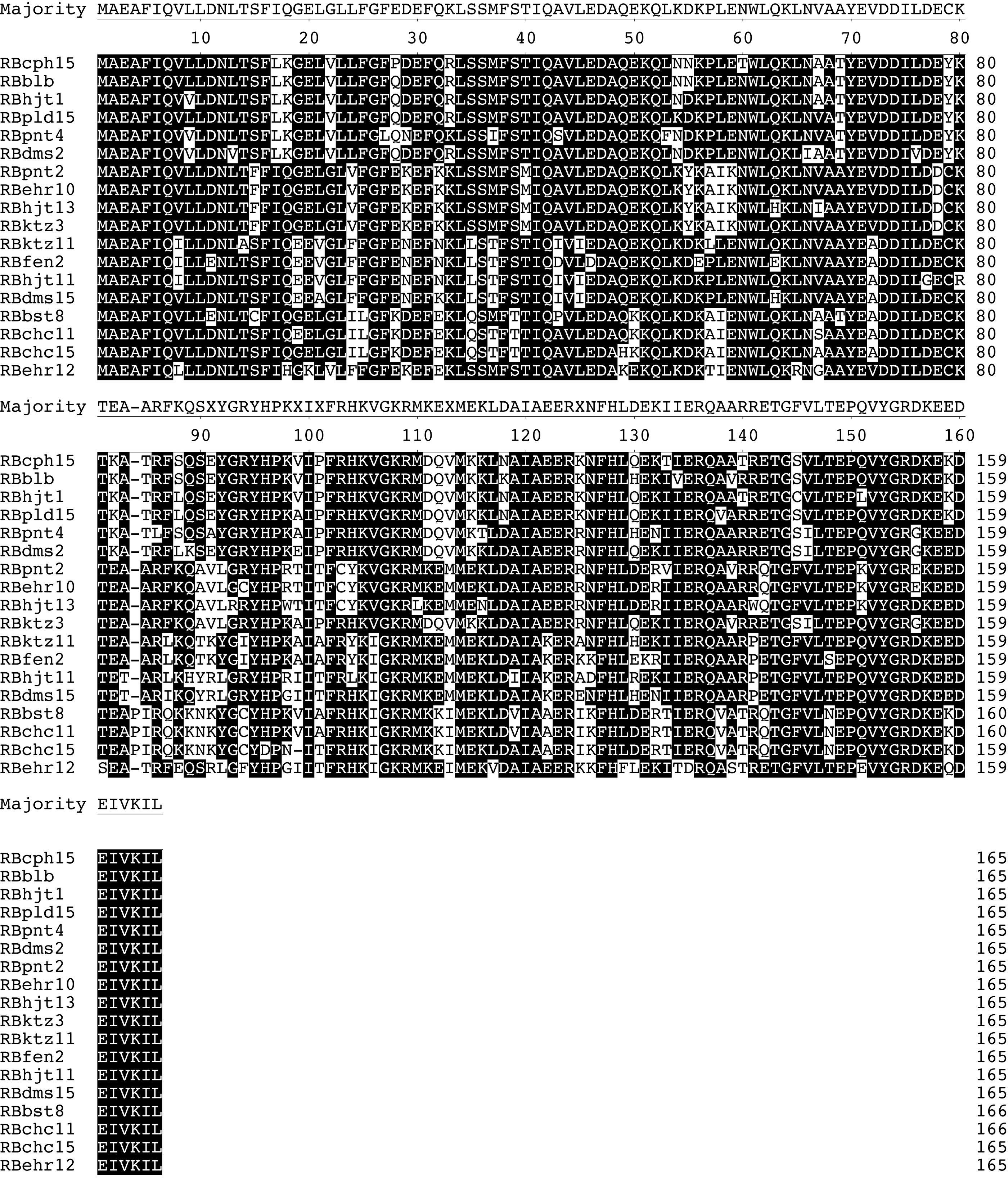

Supplement: Figure S5 — Sequence alignment of RB CC domain deduced amino acid sequences. Letters in black boxes are identical to the consensus sequence (shown at top of each row). Sequence names contain the three-letter abbreviation for each species. Numbers after the species name represents the PCR clone number. (TIF) [file ppat.1002595.s005.tif]

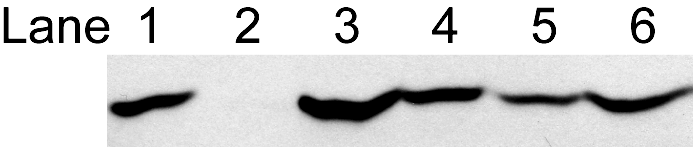

Supplement: Figure S6 — IPI-O1 mutants containing L129P are stable in RB-transgenic N. benthamiana leaves. A protein blot was performed using total protein extracts following agroinfiltration with constructs expressing indicated HA-IPI-O1 mutants. The 18-kDa protein band represents the expected size of recombinant IPI-O1 mutants. Lane1: wild type IPI-O1; Lane2: non-infiltrated N. benthamiana; Lane3: IPI-O1 L129P; Lane4: IPI-O1 Y82K/L129P; Lane5: IPI-O1 V86G/L129P; Lane6: IPI-O1 L129P/S135G. (TIF) [file ppat.1002595.s006.tif]

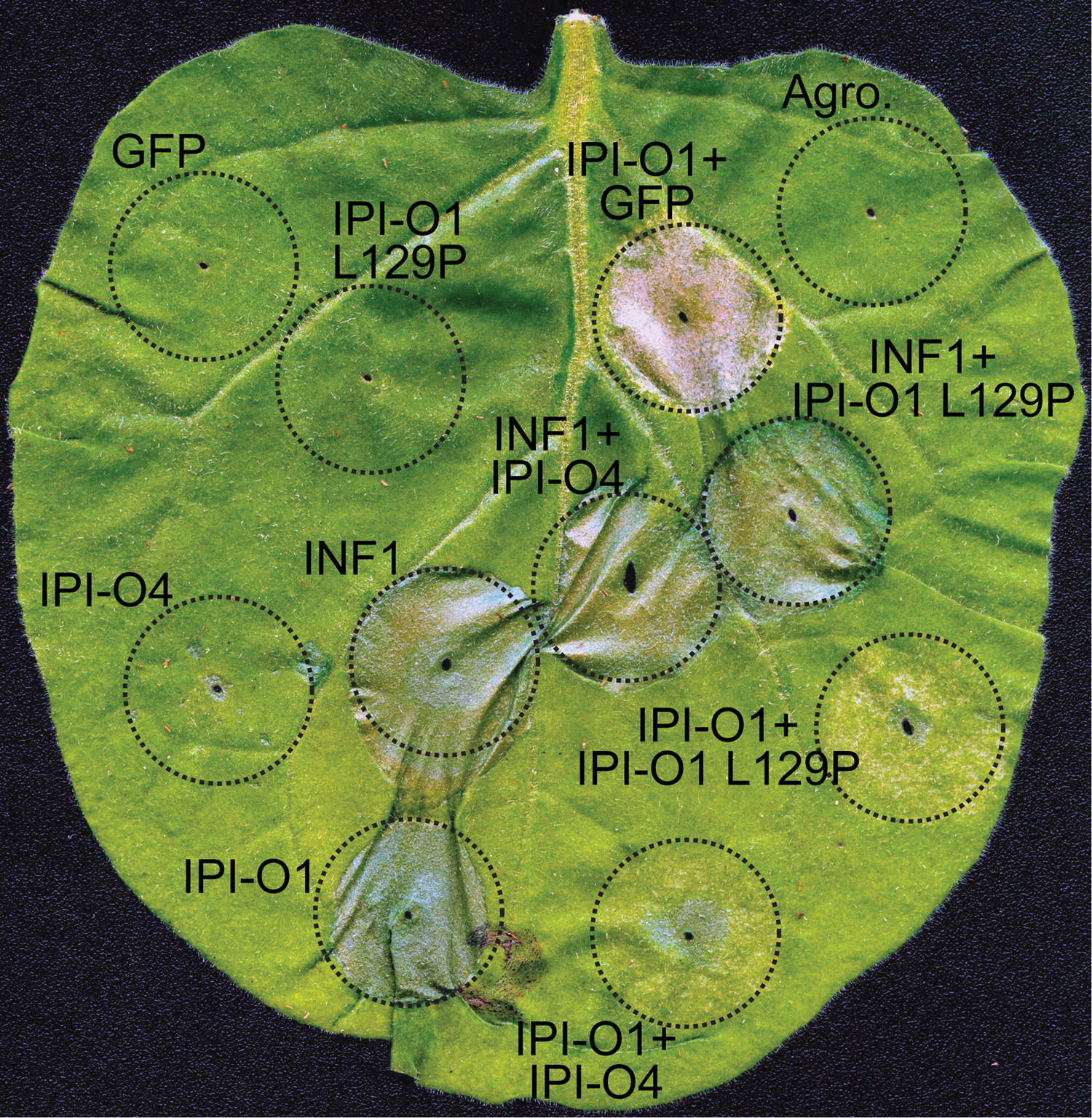

Supplement: Figure S7 — IPI-O1 L129P inhibits the HR induced by IPI-O1. A. tumefaciens strains expressing IPI-O mutants or the indicated controls were infiltrated into leaves of RB transgenic N. benthamiana plants. Leaves were photographed at 6 days after infiltration. (TIF) [file ppat.1002595.s007.tif]

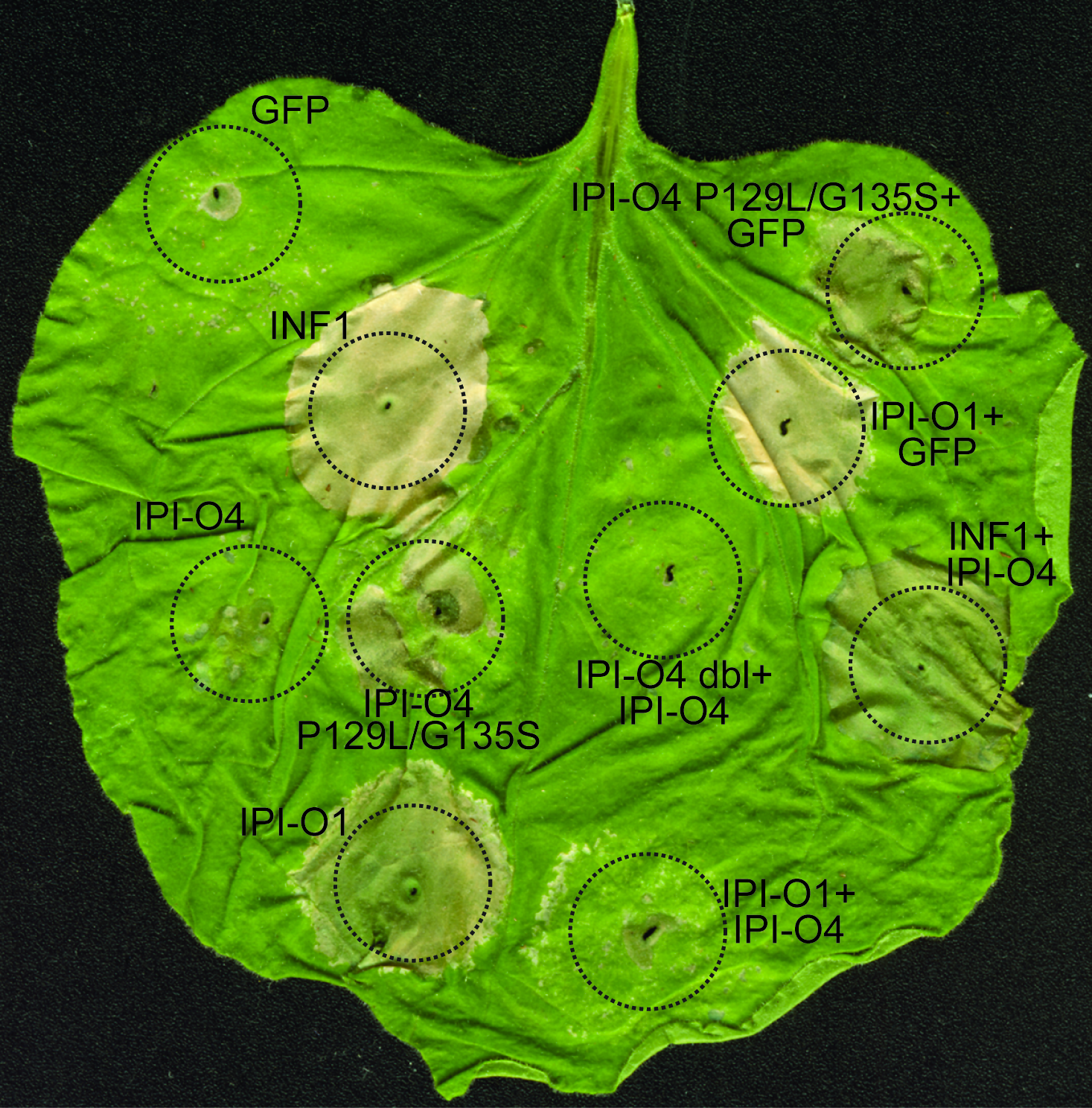

Supplement: Figure S8 — IPI-O4 inhibits the HR induced by the IPI-O4 P129L/G135S double mutant. A. tumefaciens strains expressing IPI-O mutants or the indicated controls were infiltrated into leaves of RB transgenic N. benthamiana plants. Leaves were photographed at 6 days after infiltration. (TIF) [file ppat.1002595.s008.tif]

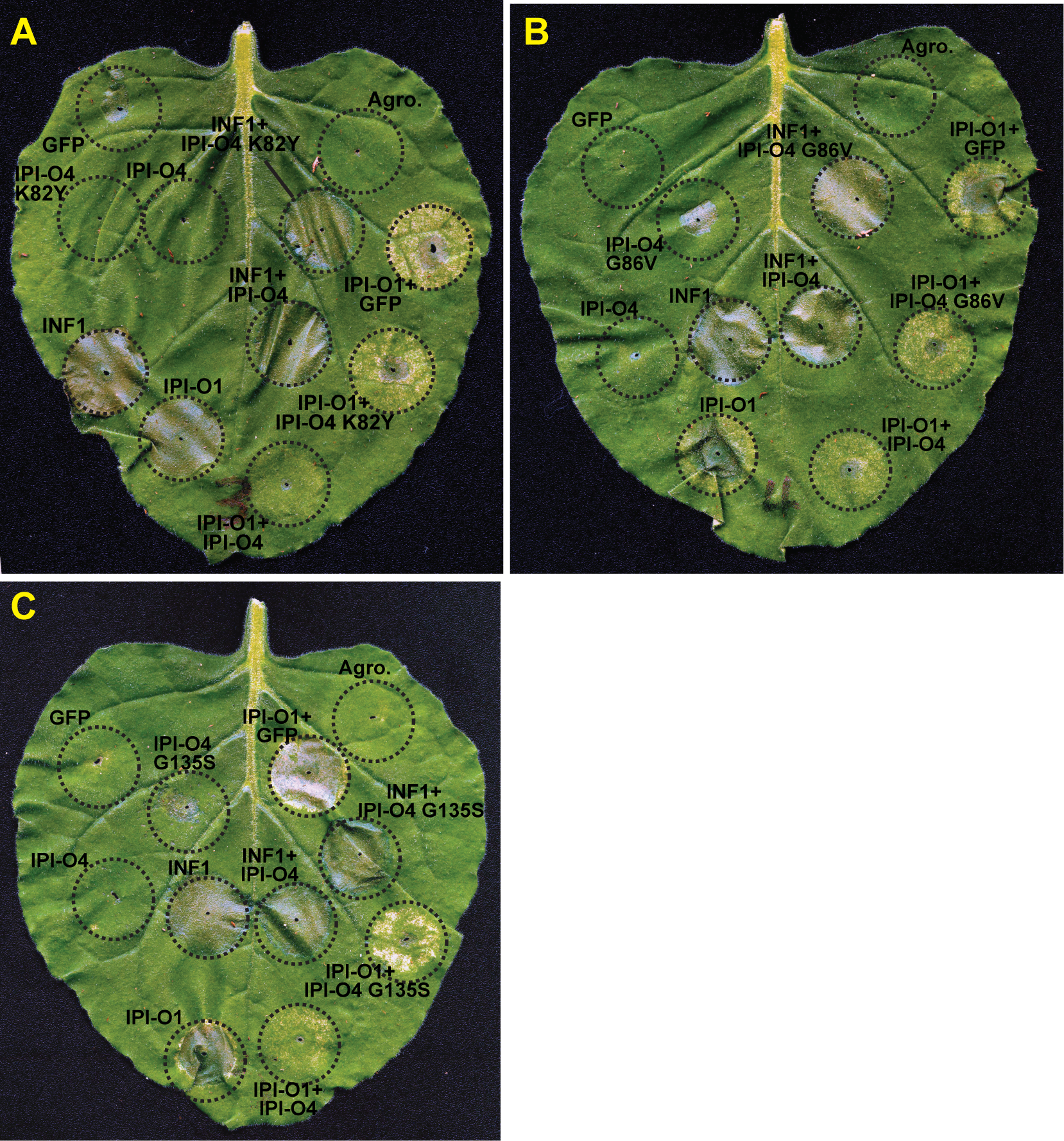

Supplement: Figure S9 — IPI-O4 K82Y, G86V, and G135S inhibit the HR induced by IPI-O1. A. tumefaciens strains expressing IPI-O mutants or the indicated controls were infiltrated into leaves of RB transgenic N. benthamiana plants. Leaves were photographed at 6 days after infiltration. Note that the inhibitory effect of these mutants is not as strong as that of IPI-O1 L129P since some cell death was still observed in the area coinfiltrated with IPI-O1 and IPI-O4 K82Y (A), G86V (B), or G135S (C). (TIF) [file ppat.1002595.s009.tif]
