# Supplementary material for: Correction: Molecular Determinants of Resistance Activation and Suppression by Phytophthora infestans Effector IPI-O
Source: PLoS Pathog. 2012 Sep 11;8(9):10.1371/annotation/75775518-f06e-4148-a639-31cfc6972b2e. doi: 10.1371/annotation/75775518-f06e-4148-a639-31cfc6972b2e (PMC3462213; doi:10.1371/annotation/75775518-f06e-4148-a639-31cfc6972b2e)
Supplement: Supplementary file 1 [file ppat.75775518-f06e-4148-a639-31cfc6972b2e.s001.docx]

Table S1. List of primers.

| **Primer Name** | **Function** | **Sequence 5’ to 3’** |
| --- | --- | --- |
| RBFORBAMHI | PCR amplification | GCCGGATCCATGGCTGAAGCTGAAGCTTTCATTC |
| RBNBFORBAMHI+ATG | PCR amplification | GCCGGATCCATGATAAACAATGTTAGTGATG |
| RBFORLRRBAMHI+ATG | PCR amplification | GCCGGATCCATGTTGGAAAAGTTTATCTCG |
| RBCCREVSALI | PCR amplification | GCCGTCGACTAGGATTTTCACTATCTCATC |
| RBNBARCREVSALI | PCR amplification | GCCGTCGACGGGGGGAAGAGTGTAAAAAAAC |
| RBREVSALI | PCR amplification | GCCGTCGACAATATATATATTCACATTAGG |
| RBFORCCECORI | PCR amplification | GCGAATTCATGGCTGAAGCTTTCATTC |
| RBFORNBARCECORI | PCR amplification | GCGAATTCATAAACAATGTTAGTGATG |
| RBFORLRRECORI | PCR amplification | GCGAATTCTTGGAAAAGTTTATCTCG |
| IPIO1FORECORI | PCR amplification | GCCGAATTCGTTTCATCCAATCTCAACACCGCCG |
| IPIO4FORECORI | PCR amplification | GCCGAATTCACCGCCGGGAATGACGCTTC |
| IPIO1FORBAMHI | PCR amplification | GCCGGATCCGTTTCATCCAATCTCAACACCGCCG |
| IPIO4FORBAMHI | PCR amplification | GCCGGATCCACCGCCGGGAATGACGCTTC |
| IPIOREVSALI | PCR amplification | GCCGTCGACAAGCTAGGGCCAACGTTTTTATC |
| IPIOREVSACI | PCR amplification | GCCGAGCTCTATACGATGTCATAGCATGAC |
| IPIO1FORBAMHI+2+ATG | PCR amplification | GCCGGATCCATATGGTTTCATCCAATCTCAACACCGCCG |
| IPIO1FORBAMH1+ATG | PCR amplification | GCCGGATCCATGGTTTCATCCAATCTCAACACCGCCG |
| IPIO4FORBAMH1+ATG | PCR amplification | GCCGGATCCATGACCGCCGGGAATGACGCTTC |
| IPIOREVXHOI+2 | PCR amplification | GCCCTCGAGAAGCTAGGGCCAACGTTTTTATC |
| IPIO1V10GFOR | Mutagenesis | AACACCGCCGGGAATTACGCT |
| IPIO1V10GREV | Mutagenesis | AGCGTAATTCCCGGCGGTGTT |
| IPIO1Y12DFOR | Mutagenesis | CGCCGTGAATGACGCTTCCAC |
| IPIO1Y12DREV | Mutagenesis | GTGGAAGCGTCATTCACGGCR |
| IPIO1S48AFOR | Mutagenesis | GCCCAACACGCTGACGAAGA |
| IPIO1S48AREV | Mutagenesis | TCTTCGTCAGCCGTGTTGGGC |
| IPIO1K58NFOR | Mutagenesis | CTATCTCAAACTCTGCGGAAT |
| IPIO1K58NREV | Mutagenesis | ATTGCGCAGAGTTTGAGATAG |
| IPIO1A60VFOR | Mutagenesis | TCAAAGTCTGTGGAATACGTG |
| IPIO1A60VREV | Mutagenesis | CACGTATTCCACAGACTTTGA |
| IPIO1Y62KFOR | Mutagenesis | GTCTGCGGAAAAAGTGAAGATGG |
| IPIO1Y62KREV | Mutagenesis | CCATCTTCACTTTTTCCGCAGAC |
| IPIO1M65LFOR | Mutagenesis | ATACGTGAAGTTGGTACTTTA |
| IPIO1M65LREV | Mutagenesis | TAAAGTACCAACTTCACGTAT |
| IPIO1V66GFOR | Mutagenesis | GTGAAGATGGGACTTTATGGA |
| IPIO1V66GREV | Mutagenesis | TCCATAAAGTCCCATCTTCAC |
| IPIO1G69AFOR | Mutagenesis | GTACTTTATGCATTCAAACTT |
| IPIO1G69AREV | Mutagenesis | AAGTTTGAATGCATAAAGTAC |
| IPIO1F70LFOR | Mutagenesis | TTTATGGATTAAAACTTGGAT |
| IPIO1F70LREV | Mutagenesis | GTACTTTATGCATTCAAACTT |
| IPIO1L721FOR | Mutagenesis | TGGATTCAAAATTGGATTTTC |
| IPIO1L721REV | Mutagenesis | GAAAATCCAATTTTGAATCCA |
| IPIO1G73AFOR | Mutagenesis | TTCAAACTTGCTTTTTCTCCTC |
| IPIO1G73AREV | Mutagenesis | GAGGAGAAAAAGCAAGTTTGAA |
| IPIO1A93YFOR | Mutagenesis | ACTGTTTACGTATCTCTATAAA |
| IPIO1A93YREV | Mutagenesis | TTTATAGAGATACGTAAACAGT |
| IPIO1Y95HFOR | Mutagenesis | TACGGCTCTCCATAAATCCGG |
| IPIO1Y95HREV | Mutagenesis | CCGGATTTATGGAGAGCCGTA |
| IPIO1 L104YFOR | Mutagenesis | GCCGAGAAGCTACAGGACCAAGC |
| IPIO1 L104YREV | Mutagenesis | GCTTGGTCCTGTAGCTTCTCGGC |
| IPIO1R105KFOR | Mutagenesis | AGAAGCCTAAAGACCAAGCAT |
| IPIO1R105KREV | Mutagenesis | ATGCTTGGTCTTTAGGCTTCT |
| IPIO1T106NFOR | Mutagenesis | AGCCTAATGAACAAGCATCTC |
| IPIO1T106NREV | Mutagenesis | GAGATGCTTGTTCCTTAGGCT |
| IPIO1L109PFOR | Mutagenesis | ACCAAGCATCCCGATAAGGCT |
| IPIO1L109PREV | Mutagenesis | AGCCTTATCGGGATGCTTGGT |
| IPIO1S115GFOR | Mutagenesis | GGCTTCCGCTGGCGTATTTTT |
| IPIO1S115GREV | Mutagenesis | AAAAATACGCCAGCGGAAGCC |
| IPIO4G10VFOR | Mutagenesis | AACACCGCCGTGAA TGACGCT |
| IPIO4G10VREV | Mutagenesis | AGCGTCATTCACGGCGG1GTT |
| IPIO4D12YFOR | Mutagenesis | CGCCGGGAATTACGCTTCCAC |
| IPIO4D12YREV | Mutagenesis | GTGGAAGCGTAATTCCCGGCG |
| IPIO4A48SFOR | Mutagenesis | GCCCAACACGTCTGACGAAGA |
| IPIO4A48SREV | Mutagenesis | TCTTCGTCAGACGTGTTGGGC |
| IPIO4N58KFOR | Mutagenesis | CTATCTCAAAGTCTGTGGAAA |
| IPIO4N58KREV | Mutagenesis | TTTCCACAGACTTTGAGATAG |
| IPIO4V60AFOR | Mutagenesis | TCAAACTCTGCGGAAAAAGTG |
| IPIO4V60AREV | Mutagenesis | CACTTTTTCCGCAGAGTTTGA |
| IPIO4K62YFOR | Mutagenesis | CTCTGTGGAATACGTGAAGTTGG |
| IPIO4K62YREV | Mutagenesis | CCAACTTCACGTATTCCACAGAG |
| IPIO4L65MFOR | Mutagenesis | AAAAGTGAAGATGGGATTGTA |
| IPIO4L65MREV | Mutagenesis | TACAATCCCATCTTCACTTTT |
| IPIO4G66VFOR | Mutagenesis | GTGAAGTTGGTATTGTATGCA |
| IPIO4G66VREV | Mutagenesis | TGCATACAATACCAACTTCAC |
| IPIO4A69GFOR | Mutagenesis | GGATTGTATGGATTAAAGATT |
| IPIO4A69GREV | Mutagenesis | AATCTTTAATCCATACAATCC |
| IPIO4L70FFOR | Mutagenesis | TGTATGCATTCAAGATTGCTT |
| IPIO4L70FREV | Mutagenesis | AAGCAATCTTGAATGCATACA |
| IPIO4172LFOR | Mutagenesis | TGCATTAAAGCTTGCTTTTTC |
| IPIO4172LREV | Mutagenesis | GAAAAAGCAAGCTTTAATGCA |
| IPIO4A73GFOR | Mutagenesis | TTAAAGATTGGATTTTCCCCAC |
| IPIO4A73GREV | Mutagenesis | GTGGGGAAAATCCAATCTTTAA |
| IPIO4Y93AFOR | Mutagenesis | ACTGTTTACGGCTCTCCATAAA |
| IPIO4Y93AREV | Mutagenesis | TTTATGGAGAGCCGTAAACAGT |
| IPIO4H95YFOR | Mutagenesis | TACGTATCTCTATAAATCCGG |
| IPIO4H95YREV | Mutagenesis | CCGGATTTATAGAGATACGTA |
| IPIO4Y104LFOR | Mutagenesis | GCCGGCTAGCCTAAAGAACAAGC |
| IPIO4Y104LREV | Mutagenesis | GCCGGCTAGCCTAAAGAACAAGC |
| IPIO4K105RFOR | Mutagenesis | GCTAGCTACAGGAACAAGCAT |
| IPIO4K105RREV | Mutagenesis | ATGCTTTGTTCCTGTAGCTAGC |
| IPIO4N106TFOR | Mutagenesis | AGCTACAAGACCAAGCATCCC |
| IPIO4N106TREV | Mutagenesis | GGGATGCTTGGTCTTGTAGCT |
| IPIO4P109LFOR | Mutagenesis | AACAAGCATCTCGATAAGGCT |
| IPIO4P109LREV | Mutagenesis | AGCCTTATCGAGATGCTTGTT |
| IPIO4G115SFOR | Mutagenesis | GGCTTCCGCTAGCGTATTTTT |
| IPIO4G115SREV | Mutagenesis | AAAAATACGCTAGCGGAAGCC |
